# *Aspergillus fumigatus* and *Aspergillus flavus*-Specific IgG Cut-Offs for the Diagnosis of Chronic Pulmonary Aspergillosis in Pakistan

**DOI:** 10.3390/jof6040249

**Published:** 2020-10-26

**Authors:** Kauser Jabeen, Joveria Farooqi, Nousheen Iqbal, Khalid Wahab, Muhammad Irfan

**Affiliations:** 1 Department of Pathology and Laboratory Medicine, Aga Khan University, Karachi 74800, Pakistan; joveria.farooqi@aku.edu (J.F.); khalid.wahab@aku.edu (K.W.); 2 Section of Pulmonary Medicine, Department of Medicine, Aga Khan University, Karachi 74800, Pakistan; nousheen.iqbal@aku.edu (N.I.); muhammad.irfan@aku.edu (M.I.); 3 Department of Medicine, Jinnah Medical and Dental College, Karachi 75400, Pakistan

**Keywords:** chronic pulmonary aspergillosis, Pakistan, *Aspergillus flavus*, *Aspergillus fumigatus*, Aspergillus IgG, Siemens immulite

## Abstract

Despite a high burden of chronic pulmonary aspergillosis (CPA) in Pakistan, *Aspergillus*-specific IgG testing is currently not available. Establishing cut-offs for *Aspergillus*-specific IgG for CPA diagnosis is crucial due to geographical variation. In settings such as Pakistan, where non-*Aspergillus fumigatus* (mainly *A. flavus*) *Aspergillus* species account for the majority of CPA cases, there is a need to explore additional benefit of *Aspergillus flavus*-specific IgG detection along with *A. fumigatus*-specific IgG detection. This study was conducted at the Aga Khan University, Karachi, Pakistan after ethical approval. Serum for IgG detection were collected after informed consent from healthy controls (*n* = 21), diseased controls (patients with lung diseases, *n* = 18), and CPA patients (*n* = 21). *A. fumigatus* and *A. flavus* IgG were detected using Siemens immulite assay. The sensitivity and specificity of *A. fumigatus*-specific IgG were 80.95% and 82.05%, respectively at a cut-off of 20 mg/L. The sensitivity and specificity of *A. flavus*-specific IgG were 80.95% and 79.49% at a cut-off of 30 mg/L. We report, for the first time, performance of *A. flavus*-specific IgG for CPA diagnosis. Although there was no statistically significant difference between the performance of both antigens, it seems contextually relevant to include *A. flavus* IgG in the CPA diagnostic algorithm in regions with higher non-*A. fumigatus* CPA infections.

## 1. Introduction

Chronic pulmonary aspergillosis (CPA) is the most common pulmonary manifestation of aspergillosis in Pakistan [1]. Significant burden of CPA as a post tuberculosis (TB) sequel has been estimated in the country [2]. A recent report from Pakistan reported TB and bronchiectasis as major underlying causes of CPA [3]. In contrast to most settings, *Aspergillus flavus* has been isolated more frequently than *Aspergillus fumigatus* from CPA cases in Pakistan [3,4]. A recent CPA guideline for use in low- and middle-income countries highlighted the use of *Aspergillus*-specific IgG as the most useful test [5]. Although several tests for detection of *Aspergillus*-specific IgG are commercially available, studies from Uganda and Europe report significant variations in cut-offs for positivity, suggesting geographical and genetic differences [6,7,8]. Currently *Aspergillus*-specific IgG testing in Pakistan is not available, highlighting a strong need to evaluate this test in the country. Establishing cut-offs for positivity of *Aspergillus*-specific IgG for CPA diagnosis in our population is also crucial to differentiate between healthy and diseased population. Additionally, as IgG-based immunoassays for CPA diagnosis have mainly been evaluated against *A. fumigatus* antigen, there is a need to explore an additional benefit of IgG detection against *A. flavus* antigen in settings such as Pakistan where non-*A. fumigatus* (mainly *A. flavus*) *Aspergillus* species account for significant proportion of CPA cases.

Therefore, in this study we aim to:

- validate the diagnostic accuracy and establish positivity cut-offs for *Aspergillus*-specific IgG using both *A. fumigatus* and *A. flavus* by testing healthy controls, diseased controls, and CPA patients using Siemens Immulite assay;
- evaluate the additional impact of detecting *A. flavus*-specific IgG in our population.

## 2. Materials and Methods

This was a prospective study (September 2019–June 2020) conducted at the Aga Khan University (AKU), Karachi, Pakistan after the approval from the AKU Ethics Review committee (ERC# 2020-1650-11613), approved on August 15, 2019. Controls of both genders were selected from healthy laboratory personnel at the clinical microbiology laboratory. CPA patients and patients with other lung diseases such as chronic obstructive pulmonary disease (COPD), asthma, interstitial lung disease (ILD), and lung cancer were recruited from pulmonology service, both outpatient and inpatient. As *Aspergillus* IgG was not available, CPA diagnosis was established using criteria from Denning et al. [5]. Allergic bronchopulmonary aspergillosis (ABPA) was diagnosed using International Society of Human and Animal Mycology (ISHAM) criteria [9].

Inclusion criteria included adults (more than 18 year of age) from both genders, who were divided into four patient populations as follows:

- healthy blood donors;
- diseased controls—diagnosed patients with COPD, asthma, ILD, lung cancer, community acquired pneumonia;
- patients with ABPA;
- patients diagnosed with CPA.

Exclusion criteria included pregnant females and immunosuppressed patients (known HIV, patients on chemotherapy, post-transplant); patients on long term steroids due to lung disease were not excluded.

A serum specimen of 5 mL was collected from both controls and patients after taking informed consent. Demographic and clinical information was collected from controls and CPA patients on a proforma. Serum was tested for *A. fumigatus* and *A. flavus*-specific IgG with Siemens Immulite assay (Siemens Healthcare Diagnostic Products Ltd., Llanberis, Gwynedd, UK) using the manufacturer’s recommendations. Immulite 2000 Allergen-Specific IgG is a solid-phase, two-step, chemiluminescent immunoassay. There is no cutoff defined by the manufacturer for positivity of each allergen. The reportable range recommended by the manufacturer is 2–200 mg/L. We did not go beyond that reportable range and the maximum level reported was >200 mg/L.

Mean ± S.D. and median with IQR of the antibody levels were calculated, and the means were compared among groups using one-way ANOVA and boxplots for the four patient populations, and t-test between CPA and non-CPA groups. ROC curve analysis was performed. Optimal diagnostic cut-offs were calculated using Youden’s J statistic (sensitivity + specificity − 1). Sensitivity and specificity were described for these cut-offs. Statistical analyses were performed using Stata version 12.1.

## 3. Results

A total of 60 individuals including 21 healthy controls, 10 diseased controls, eight ABPA, and 21 CPA patients were tested for *Aspergillus*-specific IgG using both *A. fumigatus*- and *A. flavus*-specific antigens. Description and clinical data of study participants are shown in Table 1. Radiologically, among the 21 CPA patients, 18 (85.71%) had bilateral disease. Single or multiple aspergilloma were present in 12/21 (57.14%) patients and 18/21 (85.71%) patients had cystic bronchiectasis with pleural thickening.

Antibody testing results with both antigens against different populations are shown in Table 2. Mean antibody levels for *A. flavus* and *A. fumigatus* IgG were significantly higher in CPA patients against all categories, except for ABPA patients. There was no difference in mean IgG levels between healthy and diseased controls against both antigens.

**Figure 1:**
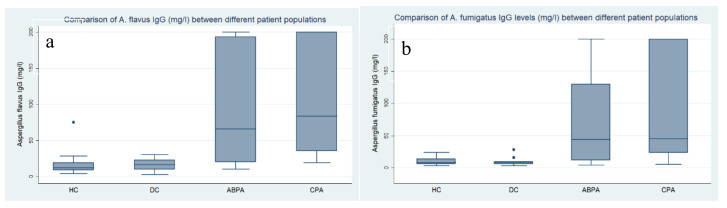
(**a**) Comparison of *A. flavus* IgG levels between categories; (**b**) Comparison of *Aspergillus fumigatus* IgG levels between categories. Note: HC, healthy controls; DC, diseased controls; ABPA, patients diagnosed with allergic bronchopulmonary aspergillosis; CPA, chronic pulmonary aspergillosis. (**a**,**b**) HC and DC had statistically significant difference in *Aspergillus*-specific IgG levels against both *A. fumigatus* and *Aspergillus flavus* allergens from CPA patients (<0.001). The difference between HC and ABPA patients was significant in *A. flavus* IgG levels (0.006) but did not reach statistical significance for *A. fumigatus* IgG.

Comparison of *A. flavus* and *A. fumigatus* IgG levels between categories is shown in Figure 1a,b.

Sensitivity, specificity, and accuracy of using different cut-offs for interpretation of IgG against *A. flavus and A. fumigatus* in CPA and non-CPA patients are shown in Table 3. The cut-off IgG levels of 30 and 20 mg/L were found to be optimal for the detection of CPA patients against *A. flavus* and *A. fumigatus* antigen, respectively. Although a cut-off of 35 mg/L for *A. flavus* had higher Youden’s statistics and a higher specificity, we considered a lower cut-off of 30 mg/L to be more sensitive for CPA case detection. The performance of different cut-offs for identifying healthy controls, diseased controls, and ABPA patients is given in Supplementary Table S1.

The data were also analyzed after excluding ABPA patients with or without CPA from our cohort. (Supplementary Tables S2 and S3). The cut-off for positivity, sensitivity, specificity, and other parameters for both antigens remained comparable to parameters reported in Table 2 and Table 3.

**Figure 2:**
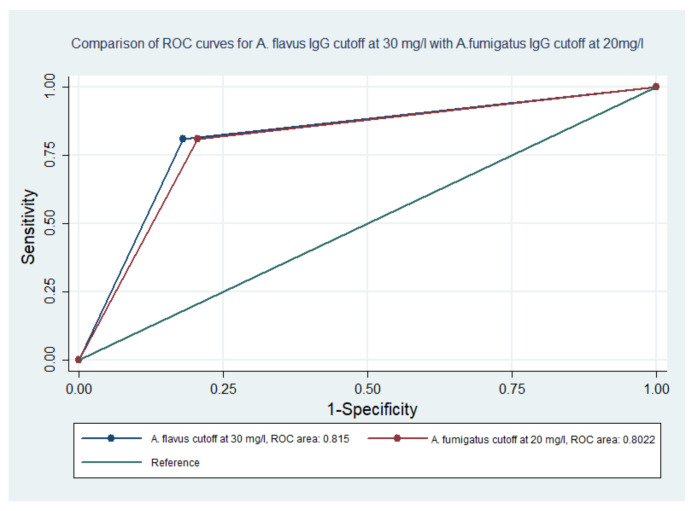
Comparison of ROC curves for *A. flavus* IgG at the cut-off of 30 mg/L and *A. fumigatus* IgG at 20 mg/L.

The comparison of ROC curves for *A. flavus* IgG at the cut-off of 30 mg/L and *A. fumigatus* IgG at 20 mg/L showed no statistically significant difference (*p*-value 0.755) (Figure 2). Similarly, testing a combination of *A. flavus* and *A. fumigatus* IgG and either *A. flavus* or *A. fumigatus* also had no difference (*p*-values of 0.951 and 0.951, respectively). Four CPA patients had IgG levels less than 10 mg/L when tested with *A. fumigatus* and had IgG levels ranging from 20 to 53.3 mg/L with *A. flavus*. Among these patients, one patient received itraconazole followed by voriconazole for one year and had IgG levels of 5.38 and 20 mg/L, respectively, against *A. fumigatus* and *A. flavus*. Another patient received itraconazole for six months and had IgG levels of 9.34 and 53.3 mg/L, respectively, against *A. fumigatus* and *A. flavus*. Two patients did not receive any prior antifungal treatment and had completely fibrosed hemithorax. These patients had IgG levels of 9.28 and 7.85 against *A. fumigatus* and 24.1 and 21.0 mg/L against *A. flavus.*

## 4. Discussion

This is the first report from Pakistan establishing the optimal cut-off and performance of *A. fumigatus* IgG levels for CPA diagnosis. Additionally, to the best of our knowledge, this is the first evaluation of performance of *A. flavus* IgG for CPA diagnosis using newer immunoassays. Estimation of *A. flavus* IgG levels is especially relevant in settings where non-*fumigatus Aspergillus* species are a common cause of pulmonary infections.

*A. flavus* is the most prevalent species across all clinical spectra of aspergillosis in Pakistan [1,3,4]. A high concentration of *A. flavus* has also been reported from the country in both indoor and outdoor environments [10,11]. Therefore, we also determined *A. flavus* IgG levels in all of our study participants to evaluate its performance, as well as its incremental value in CPA diagnosis along with *A. fumigatus* IgG. The performance of *A. flavus* IgG was found to be similar to *A. fumigatus* IgG with comparable sensitivity and specificity. Mean and median *A. flavus* IgG levels were higher than *A. fumigatus* IgG in both healthy and diseased controls, as well as CPA patients. The cut-off for CPA diagnosis was also higher for *A. flavus* IgG than *A. fumigatus* (30 mg/L vs. 20 mg/L, respectively). We hypothesize this difference to be due to continuous exposure of the local population to *A. flavus* spores in the environment leading to development of IgG antibodies. Our result also showed no incremental value of detecting both *A. flavus* and *A. fumigatus* IgG level. However, one of the four patients, who had an IgG level less than 10 mg/L using *A. fumigatus* allergen, had an IgG level of 56 mg/L against *A. flavus,* despite receiving itraconazole for the past six months; other remaining patients also had higher levels than 20 mg/L for *A. flavus*-specific IgG. Another patient, with *A. fumigatus* IgG level less than 10 mg/L, had been receiving itraconazole for the past year, however, *A. flavus* IgG level was 20 mg/L. We suggest that detection of *A. flavus* IgG is more useful for CPA diagnosis in our setup, however more data are required to make a stronger recommendation.

Most of the studies that have evaluated the performance of *Aspergillus* IgG for CPA diagnosis have used *A. fumigatus* as an allergen. We did not come across any study that had evaluated *A. flavus* IgG by ELISA-based assays. However, a study from Taiwan evaluated both *A. fumigatus* and *Aspergillus niger* IgG to determine seroprevalence and CPA prevalence. They also reported a high correlation between *A. fumigatus* and *A. niger* IgG levels [12].

The median of *A. fumigatus* IgG of healthy controls in our study was found to be 7.6 mg/L. This value is higher than healthy controls from Uganda, lower than European healthy controls, and closer to healthy controls from Indonesia [13,14]. The median of *A. fumigatus* IgG levels of diseased controls was 8.5 mg/L, comparable to that reported previously [13,14].

Regarding *A. fumigatus* IgG, a cut-off of 20 mg/L for CPA patents was found to be optimal with a sensitivity and specificity of 80.95 and 79.49, respectively. This cut-off is similar to that reported from UK and Uganda in CPA patients and lower than that reported from Indonesia using similar Immulite assay [7,13,14]. Our results confirmed geographical variation in the optimal cut-off for CPA diagnosis.

The sensitivity and specificity of *A. fumigatus*-specific IgG, reported in our study, is lower than that reported in previous studies evaluating similar assay [6,7,13,14]. Another study from India, a neighboring country to Pakistan with a similar population, evaluating ImmunoCAP assay also reported higher sensitivity and specificity [15]. The preferable assay for CPA diagnosis has been proposed to have sensitivity and specificity of more than 90% and 85%, respectively [5]. The highest sensitivity of *A. fumigatus* IgG, achieved in our study, was 80.95% at the cut-offs of 10, 15 and 20 mg/L. The sensitivity of *A. flavus* IgG, however, increased to 95.2% at the cut-off of 20 mg/L. However, the specificity and the accuracy were greatly compromised. The probable reasons for the lower sensitivity in our study could be a low sample size, and we assume that accuracy of the test would increase in a larger sample. Our study population of CPA patients had different associated conditions and were not limited to post TB only. The patients were also at variable stages of CPA. We conducted our study on a non-homogenous sample to represent the actual patient population that presents to pulmonary care services.

In ABPA patients, there is a persistence of inhaled *Aspergillus* species conidia which germinate and elicit an immune response and lead to sustained inflammation and tissue damage [9]. Levels of both *Aspergillus*-specific IgE and IgG are elevated in these patients [9]. Additionally, these patient also develop CPA represented as Aspergillus overlap syndrome [16]. Our study identified that detection of *Aspergillus* IgG in ABPA patients is not helpful to diagnose progression of these patients towards CPA. This knowledge is especially relevant in settings where CPA is still an under recognized entity. In such settings physicians should be educated that, in patients with ABPA, CPA diagnosis should not rely on detection of *Aspergillus* IgG levels and alternative diagnostic modalities should be used.

Our study has many limitations. First, there was a low sample size in all study groups, however this was an initial study and we plan to collect more data. We did not compare the results of Siemen’s immulite assay with any other platform. It was a single center study limited to patients from Karachi. The study population included was non-homogenous to represent real-life patient referral, and CPA patients were at various stages of disease and antifungal treatment. Despite all of these limitations, this is the first study from Pakistan, evaluating a diagnosis for a prevalent condition. We also evaluated, for the first time, the performance of *A. flavus*-specific IgG, as this species is more commonly isolated from patients with aspergillosis in Pakistan.

## 5. Conclusions

We report sensitivity and specificity of 80.95% and 82.05%, respectively, at a cut-off of 20 mg/L of *A. fumigatus*-specific IgG. The sensitivity and specificity of *A. flavus*-specific IgG were 80.95% and 79.49% at a cut-off of 30 mg/L. Although there was no statistically significant difference between the performance of both allergens-specific IgGs, it seems contextually relevant to include *A. flavus* IgG in the CPA diagnostic algorithm in regions with higher non-*A. fumigatus* CPA infections.

## Figures and Tables

**Table 1:** Description and clinical data of study participants.

	Healthy Control = 21	Diseased Control = 18	CPA Patients = 21
Gender (M/F)	11/10	14/4	12/9
Mean age in years	32	47	44
Associated lung disease			
ABPA		8	3
COPD		3	5
Asthma		10	4
Bronchiectasis		3	15
Post TB		2	10
Other		1	5
Antifungal treatment			17
Itraconazole			13
Voriconazole			4
No antifungal therapy			4

ABPA: Allergic bronchopulmonary aspergillosis; COPD: Chronic obstructive pulmonary disease.

**Table 2:** Antibody testing results with both antigens against different populations.

	*n*	Mean mg/L	SD	Median	Range	95% Centile	IQR	Mean Difference from Patient Category: mg/L (*p*-Value)
***Aspergillus flavus* IgG**								
HC	21	13.2	6.1	11.9	4.3–28.6	23.1	9.0–15.1	DC: 9.1 (1.00) ABPA: 83.0 (0.006) * CPA: 96.5 (<0.001) *
DC **	10	22.3	20.8	16.6	2.8–75.6	75.6	9.9–28.3	ABPA: 73.9 (0.055) CPA: 87.4 (0.001) *
ABPA	8	96.2	86.7	66.1	10.3–200	200	20.1–193.5	CPA: 13.5 (1.00)
Non-CPA#	39	32.6	50.9	15.1	2.8–200	200	9.9–24.6	CPA: 77.1 (<0.001) *
CPA	21	109.7	80.2	83.9	19.5–200	200	35.6–200	-
***Aspergillus fumigatus* IgG**								
HC	21	9.0	4.9	7.6	3.0–21.1	19.4	5.7–10.8	DC: 2.5 (1.00) ABPA: 62.8 (0.057) CPA: 76.8 (<0.001) *
DC	10	11.6	8.3	8.5	3.1–27.8	27.8	5.7–15.9	ABPA: 60.3 (0.168) CPA: 74.2 (0.007) *
ABPA	8	71.8	81.5	43.8	3.9–200	200	11.4–130.1	CPA: 13.9 (1.00)
Non-CPA #	39	22.6	43.6	8.28	3.1–200	200	5.7–15.9	CPA: 63.2 (0.001)
CPA	21	85.8	80.6	45.1	5.4–200	200	23.1–200	-

Note: HC, healthy controls; DC, diseased controls. * *p*-value < 0.05 and ** DC excluding ABPA patients, # Non-CPA category includes HC, DC, and ABPA patients.

**Table 3:** Sensitivity, specificity, and accuracy of using different cut-offs for interpretation of *Aspergillus* IgG in CPA patients.

*Aspergillus flavus* CPA Patients: 21							
Cut-Off (mg/L)	No. of CPA Patients above This Cut-Off	Sensitivity	Specificity	Accuracy	ROC AUC	95% CI	Youden’s J Statistics
10	21	100	28	53	0.64	0.50–0.75	-
15	21	100	47	67	0.74	0.62–0.85	-
20	20	95	64	75	0.80	0.68–0.89	0.593
25	18	86	77	80	0.81	0.70–0.90	0.626
**30**	**17**	**81**	**82**	**82**	**0.82**	**0.70–0.90**	**0.630**
**35**	**16**	**76**	**87**	**83**	**0.82**	**0.70–0.90**	**0.634**
40	13	62	87	78	0.75	0.62–0.85	0.491
***Aspergillus fumigatus* CPA Patients: 21**							
**Cut-Off** **(mg/L)**	**No. of Patients above This Cut-Off**	**Sensitivity**	**Specificity**	**Accuracy**	**ROC AUC**	**95% CI**	**Youden’s J Statistics**
10	17	81	61.5	68	0.71	0.59–0.83	0.425
15	17	81	74	77	0.78	0.66–0.88	0.553
**20**	**17**	**81**	**79**	**80**	**0.80**	**0.68–0.89**	**0.604**
25	15	71	85	80	0.78	0.66–0.88	0.560
30	13	62	87	78	0.75	0.62–0.85	0.491
35	13	62	87	78	0.75	0.62–0.85	0.491
40	11	52	90	77	0.71	0.59–0.83	0.421

Bold values represent proposed cut-offs with highest Youden’s statistics.

## Supplementary Materials

The following are available online at https://www.mdpi.com/2309-608X/6/4/249/s1, Table S1: Sensitivity, specificity, and accuracy of using different cut-offs for interpretation of *Aspergillus* IgG in healthy controls, diseased controls, and ABPA patients. Table S2: Antibody testing results with both allergens against different populations after excluding ABPA patients with or without CPA from the analysis; Table S3: Sensitivity, specificity, and accuracy of using different cut-offs for interpretation of *Aspergillus* IgG in CPA patients after excluding ABPA patients with or without CPA from the analysis.
