# Supplementary material for: Aspergillus fumigatus and Aspergillus flavus-Specific IgG Cut-Offs for the Diagnosis of Chronic Pulmonary Aspergillosis in Pakistan
Source: J Fungi (Basel). 2020 Oct 26;6(4):249. doi: 10.3390/jof6040249 (PMC7711809; doi:10.3390/jof6040249)
Supplement: Supplementary file 1 [file jof-06-00249-s001.pdf]

Supplementary table 1: Sensitivity, specificity and accuracy of using different cut-offs for interpretation of *Aspergillus* IgG in healthy controls, diseased controls and ABPA patients

Aspergillus flavus **healthy controls: 21**							
**Cut-off (mg/L)**	**No. of controls at or below this cut-off**	**Sensitivity**	**Specificity**	**Accuracy**	**ROC AUC**	**95%CI**	**Youden's J statistics**
10	8	38.10	92.31	73.33	0.65	0.51-0.77	0.304
15	15	71.43	89.74	83.33	0.81	0.68-0.89	0.612
20	17	80.95	76.92	78.33	0.79	0.66-0.88	0.579
**25**	**20**	**95.24**	**66.67**	**76.67**	**0.81**	**0.70-0.90**	**0.619**
30	21	100.00	61.54	75.00	0.81	0.68-0.89	-
35	21	100.00	53.85	70.00	0.75	0.64-0.87	-
40	21	100.00	46.15	65.00	0.73	0.60-0.84	-
Aspergillus flavus **diseased controls: 10**							
**Cut-off (mg/L)**	**No. of controls at or below this cut-off**	**Sensitivity**	**Specificity**	**Accuracy**	**ROC AUC**	**95%CI**	**Youden's J statistics**
10	3	30.00	84.00	75.00	0.57	0.43-0.69	0.140
15	3	30.00	68.00	61.67	0.49	0.35-0.62	0.020
20	6	60.00	60.00	60.00	0.60	0.47-0.72	0.200
25	7	70.00	48.00	51.67	0.59	0.45-0.71	0.180
30	8	80.00	44.00	50.00	0.62	0.48-0.74	0.240
**35**	**9**	**90.00**	**40.00**	**48.33**	**0.65**	**0.52-0.77**	**0.300**
40	9	90.00	34.00	43.33	0.62	0.48-0.74	0.240
Aspergillus flavus **ABPA patients: 8**							
**Cut-off (mg/L)**	**No. of ABPA patients at or below this cut-off**	**Sensitivity**	**Specificity**	**Accuracy**	**ROC AUC**	**95%CI**	**Youden's J statistics**
10	8	100.00	21.15	31.67	0.60	0.47-0.72	-
15	7	87.50	34.62	41.67	0.61	0.48-0.74	0.222
20	6	75.00	46.15	50.00	0.61	0.47-0.72	0.211
25	5	62.50	57.69	58.33	0.60	0.47-0.72	0.202
**30**	**5**	**62.50**	**63.46**	**63.33**	**0.63**	**0.50-0.75**	**0.260**
35	4	50.00	67.31	65.00	0.59	0.45-0.71	0.173
40	4	50.00	73.08	70.00	0.62	0.48-0.74	0.231
Aspergillus fumigatus **healthy controls: 21**							
**Cut-off (mg/L)**	**No. of controls at or below this cut-off**	**Sensitivity**	**Specificity**	**Accuracy**	**ROC AUC**	**95%CI**	**Youden's J statistics**
10	15	71.43	66.67	68.33	0.69	0.55-0.80	0.381

15	19	80.48	64.10	73.33	0.77	0.64-0.87	0.546
**20**	**20**	**95.24**	**61.54**	**73.33**	**0.78**	**0.66-0.88**	**0.568**
25	21	100.00	53.85	70.00	0.77	0.64-0.87	-
30	21	100.00	46.15	65.00	0.73	0.60-0.84	-
35	21	100.00	46.15	65.00	0.73	0.60-0.84	-
40	21	100.00	38.46	60.00	0.69	0.57-0.81	-
***Aspergillus fumigatus* diseased controls: 10**							
**Cut-off (mg/L)**	**No. of controls at or below this cut-off**	**Sensitivity**	**Specificity**	**Accuracy**	**ROC AUC**	**95%CI**	**Youden's J statistics**
10	7	70.00	58.00	60.00	0.64	0.50-0.75	0.280
15	7	70.00	48.00	**51.67**	0.59	0.45-0.71	0.180
20	8	80.00	46.00	51.67	0.63	0.50-0.75	0.260
**25**	**8**	**90.00**	**40.00**	**48.33**	**0.65**	**0.52-0.77**	**0.300**
30	9	100.00	36.00	46.67	0.68	0.55-0.80	-
35	9	100.00	36.00	46.67	0.68	0.55-0.80	-
40	9	100.00	30.00	41.67	0.65	0.52-0.77	-
***Aspergillus fumigatus* ABPA patients: 8**							
**Cut-off (mg/L)**	**No. of ABPA patients at or below this cut-off**	**Sensitivity**	**Specificity**	**Accuracy**	**ROC AUC**	**95%CI**	**Youden's J statistics**
10	6	75.00	50.00	53.33	0.63	0.50-0.75	0.250
15	5	62.50	57.69	58.33	0.60	0.47-0.72	0.202
20	5	62.50	61.54	61.67	0.62	0.48-0.74	0.240
25	5	62.50	69.23	68.33	0.66	0.53-0.78	0.317
**30**	**5**	**62.50**	**75.00**	**73.33**	**0.69**	**0.55-0.80**	**0.375**
35	5	62.50	75.00	73.33	0.69	0.55-0.80	0.375
40	4	50.00	78.85	75.00	0.64	0.51-0.77	0.288

Note: HC: Healthy controls, DC: Diseased controls, *p-value <0.05, **DC excluding ABPA patients, #Non-CPA category includes HC, DC and ABPA patients.

Supplementary Table 2. Antibody testing results with both allergens against different populations after excluding ABPA patients with or without CPA from the analysis.

	n	Mean mg/L	SD	Median	Range	95% centile	IQR	Mean difference from patient category: mg/L (*p*-value)
Aspergillus flavus **IgG**								
Healthy controls (HC)	21	13.2	6.1	11.9	4.3–28.6	23.1	9.0–15.1	DC: 9.1 (1.00)<br>CPA: 81.5 (<0.001)*
Diseased Controls (DC)**	10	22.3	20.8	16.6	2.8–75.6	75.6	9.9–28.3	CPA: 72.4 (0.001)*
Non-CPA#	31	16.2	13.2	13.3	2.8–75.6	30.5	9.0–20.0	CPA: 78.5 (0.0004)*
**CPA**	**18**	**94.8**	**76.8**	**53.8**	**19.5–200**	**200**	**30.6–200**	-
Aspergillus fumigatus **IgG**								
Healthy controls (HC)	21	9.0	4.9	7.6	3.0–21.1	19.4	5.7–10.8	DC: 2.5 (1.00)<br>CPA: 65.9 (<0.001)*
Diseased Controls (DC)	10	11.6	8.3	8.5	3.1–27.8	27.8	5.7–15.9	CPA: 63.3 (<0.001)*
Non-CPA#	31	9.8	6.2	7.7	3.0–27.8	23.7	5.7–13.6	CPA: 65.0 (<0.0001)
**CPA**	**18**	**74.9**	**80.9**	**38.1**	**5.4–200**	**200**	**20.8–200**	**-**

Supplementary Table 3. Sensitivity, specificity, and accuracy of using different cut-offs for interpretation of *Aspergillus* IgG in CPA patients after excluding ABPA patients with or without CPA from the analysis.

Aspergillus flavus **CPA patients: 18**							
**Cut-off (mg/L)**	**No. of CPA patients above this cut-off**	**Sensitivity**	**Specificity**	**Accuracy**	**ROC AUC**	**95%CI**	**Youden's J statistics**
10	18	100	35	59	0.68	0.52–0.80	-
15	18	100	58	73	0.79	0.66–0.90	-
20	17	94	74	81	0.84	0.70–0.93	0.686

25	15	83	87	86	0.85	0.73–0.94	0.704
**30**	**14**	**78**	**93.5**	**88**	**0.86**	**0.72–0.94**	**0.713**
**35**	**13**	**72**	**97**	**88**	**0.85**	**0.70–0.93**	**0.690**
40	10	55.5	97	81	0.76	0.61–0.87	0.523
Aspergillus fumigatus **CPA patients: 18**							
**Cut-off (mg/L)**	**No. of patients above this cut-off**	**Sensitivity**	**Specificity**	**Accuracy**	**ROC AUC**	**95%CI**	**Youden's J statistics**
10	14	78	71	73	0.74	0.59–0.85	0.487
15	14	78	84	82	0.81	0.68–0.91	0.616
**20**	**14**	**78**	**90**	**86**	**0.84**	**0.70–0.93**	**0.681**
25	12	67	97	87	0.82	0.68–0.91	0.634
30	10	55.5	100	84	0.78	0.63–0.88	-
35	10	55.5	100	84	0.78	0.63–0.88	-
40	8	44	100	79.5	0.72	0.57–0.83	-
